# Risk of adverse perinatal outcomes among women with clinical and subclinical histopathological chorioamnionitis

**DOI:** 10.3389/fmed.2024.1242962

**Published:** 2024-03-06

**Authors:** Andrea Olguín-Ortega, Ricardo Figueroa-Damian, Martha Leticia Palafox-Vargas, Enrique Reyes-Muñoz

**Affiliations:** ^1^Department of Gynecology, National Institute of Perinatology, Mexico City, Mexico; ^2^Department of Infectology, National Institute of Perinatology, Mexico City, Mexico; ^3^Department of Pathology, National Institute of Perinatology, Mexico City, Mexico; ^4^Coordination of Gynecological and Perinatal Endocrinology, National Institute of Perinatology, Mexico City, Mexico

**Keywords:** chorioamnionitis, pregnancy outcome, placenta, premature birth, stillbirth

## Abstract

**Introduction:**

Histologic chorioamnionitis (HCA) is a placental inflammatory condition associated with adverse perinatal outcomes (APOs). This historical cohort study explores the risk of APOs in pregnant women with HCA and compares the impact of clinical chorioamnionitis (CCA) with subclinical chorioamnionitis (SCCA).

**Methodology:**

Placentas were evaluated by a perinatal pathologist tand all women with HCA were included. Two groups were integrated: (1) women with clinical chorioamnionitis (CCA) and (2) women with subclinical chorioamnionitis (SCCA). Additionally, we conducted a secondary analysis to compare the prevalence of APOs among stage 1, 2 and 3 of HCA and the risk of APOs between grades 1 and 2 of HCA. The APOs analyzed were preterm birth, stillbirth, neonatal weight < 1,500 g, neonatal sepsis. Relative risk with 95% confidence interval was calculated.

**Results:**

The study included 41 cases of CCA and 270 cases of SCCA. The mean gestational age at diagnosis and birth was 30.2 ± 5.4 weeks and 32.5 ± 5.1 weeks, for group 1 and 2, respectively. The study also found that women with HCA stage 3 and grade 2 had a higher prevalence and risk of adverse perinatal outcomes.

**Discussion:**

The findings of this study suggest the importance of placental histological study to excluded SCCA, which represents a significant risk to both maternal and neonatal health, contributing to high morbidity and mortality.

## 1 Introduction

Preterm birth (PTB), which occurs in about 12% of pregnancies worldwide, is the main cause of neonatal morbidity and mortality (1). PTB is a syndrome that can be precipitated by various factors, including infection, cervical pathology, uterine overdistension, progesterone deficiency, vascular alterations (such as uteroplacental ischemia and decidual abruption, maternal and fetal stress, maternal-fetal alloimmune response, allergic phenomena, and likely other undetermined factors) (2). While the intra-amniotic inflammatory responses driven by microbes (infection) or alarmins (sterile) have some overlap in the participating cellular and molecular processes, the distinct natures of these two conditions necessitate the implementation of specific approaches to prevent adverse pregnancy and neonatal outcomes (3). Almost half of all preterm births are caused or triggered by an inflammatory process at the feto-maternal interface resulting in preterm labor or rupture of membranes with or without chorioamnionitis (“first inflammatory hit”) (4).

Acute chorioamnionitis is characterized by neutrophilic infiltration and inflammation at the maternal fetal interface (5). Chorioamnionitis encompasses an heterogeneous setting of conditions characterized by infection or inflammation or both, followed by a great variety in clinical practice for mothers and their newborns (6). Placental inflammation is often clinically silent and can signal the normal physiologic process of parturition, an inflammatory process, but can also be a sign of sub-clinical infection (7).

Typically, the clinical presentation of chorioamnionitis is defined as acute chorioamnionitis (8). Histologic chorioamnionitis (HCA) is defined as an intrauterine inflammatory condition characterized by acute granulocyte infiltration into the fetal–maternal or the fetal tissues, the prevalence of HCA is inversely correlated with gestational age, occurring in 50% of PTB and in up to 20% of deliveries at term (9). Correlations exist between the severity of histological maternal/fetal inflammatory responses and the prevalence of clinical chorioamnionitis (CCA) and positive maternal clinical signs in preterm deliveries, however, the prevalence of CCA has been reported as 20 to 30%, even in cases characterized by the most severe fetal inflammatory responses (10).

HCA is defined as acute inflammatory lesions of the placenta consisting of diffuse infiltration of neutrophils at different sites in the organ (11). The Perinatal Section of the Society of Pediatric Pathology provided a template to classify histopathologic chorioamnionitis according to the findings (12), the actual classification system by the Amsterdam Placental Workshop Group Consensus Statement delineates stages and grades for maternal inflammatory responses in the placenta:

1. Stage 1—Acute Subchorionitis or Chorionitis: Initial inflammation within the chorion.
2. Stage 2—Acute Chorioamnionitis: Inflammation in the chorioamnion, reaching into the fibrous chorion and/or amnion.
3. Stage 3—Necrotizing Chorioamnionitis: Severe inflammation with cellular damage and necrosis in the amnion and chorion.

For each stage:

- Grade 1: Not defined as severe.
- Grade 2: Severe inflammation, possibly with microabscesses or confluent leukocytes (13).

The accuracy of CCA in detecting intra-amniotic infection (IAI) is around 50%; the accuracy of each diagnostic criterion to diagnose IAI is 51.1% with maternal tachycardia, 57.8% with fetal tachycardia, and 55.6% with maternal leukocytosis, however, it is important to note that these diagnostic performances were obtained from term pregnancies and not from preterm pregnancies (14).

CCA is a relatively common complication of pregnancy and can have devastating consequences including preterm labor, maternal infections, fetal infection/inflammation, fetal lung, brain, and gastrointestinal tract injury (5). The confirmed sepsis rates were 7% (early-onset) and 22% (late-onset) for the histological group, and 6% (early-onset) and 26% (late-onset) for CCA-exposed neonates (15). In other reports, a meta-analysis showed an association between any type of CA and any early onset sepsis (OR 4.29, CI 3.63–5.06), any late onset sepsis (OR 1.29, CI 1.11–1.54) (15), and any undetermined onset sepsis (OR 1.59, CI 1.11–1.54) (16). Peripartum infection pooled incidence in high-quality studies has been reported to be 3.9% (95% Confidence Interval [CI] 1.8–6.8%) for chorioamnionitis, 1.6% (95% CI 0.9–2.5%) for endometritis, 1.2% (95% CI 1.0–1.5%) for wound infection, 0.05% (95% CI 0.03–0.07%) for sepsis, and 1.1% (95% CI 0.3–2.4%) for maternal peripartum infection (17).

The impact of SCCA has been described in several studies, and it is associated with an increased risk of PTB with prelabor rupture of membranes (PROM) (aRR: 3.92 (95% CI: 2.15, 7.12)) and early PTB (aRR: 1.77 (95% CI, 1.18, 2.64)) (18). Historically, it has been very difficult to diagnose without histologic examination, the detection of inflammatory biomarkers in the amniotic fluid by surface-enhanced laser desorption/ionization time-of-flight mass spectroscopy has been proposed since it has exhibited high diagnostic accuracy for SCCA (19). Additionally, the combined measurements of maternal nuclear factor kappa B-p65 and C-reactive protein levels may be used as early biological indicators that predict SCCA in premature rupture of membranes (20).

Since 2017, the American College of Obstetricians and Gynecologists (ACOG) proposed that the diagnosis of suspected IAI was made when the maternal temperature was greater than or equal to 39.0°C or when the maternal temperature is 38.0 to 38.9°C and an additional clinical risk factor is present (maternal leukocytosis, purulent cervical drainage, or fetal tachycardia) (21). Among women who met old ACOG criteria for IAI, but not the new criteria, postpartum infection occurs in nearly 10% (22).

It is important to highlight that the fetus possesses the ability to initiate an inflammatory response, either at a local or systemic level, upon exposure to microorganisms or non-infectious stimuli (such as danger signals or alarmins), the Fetal Inflammatory Response Syndrome (FIRS) was coined to characterize a condition characterized by signs of a systemic inflammatory response, frequently triggered by the activation of the innate immune response (23). Histopathology, while not immediately addressing pressing clinical inquiries, warrants consideration due to its enduring significance; its findings hold the potential to evaluate the lifelong risks associated with a broad spectrum of diseases linked to prenatal exposures such as FIRS (24).

The aim of this study was to evaluate the risk of adverse perinatal outcomes in women with HCA with CCA compared to SCCA.

## 2 Materials and methods

### 2.1 Study design and participants

A historical cohort study of consecutive women diagnosed with HCA attended at the National Institute of Perinatology, Mexico City, from January 2007 to December 2011. The Institutional Review Board and Ethics Committee approved this study with registration number: CEI-RETRO-01-2023.

The National Institute of Perinatology is a public tertiary-level healthcare facility that provides medical attention to low-income patients, with high-risk pregnancies from the metropolitan area of the Valley of Mexico. During the study period, were attended at the Institute 5,500 deliveries per year, with a rate of prematurity of 18%, preeclampsia rate of 6% and frequency of neonates small for gestational age of 14%. The inclusion criteria were women with HCA (placental histological lesions diagnosed by an experienced perinatal pathologist). The severity of—HCA was determined according to the Amsterdam Placental Workshop Group Consensus Statement (13). The exclusion criteria were women with severe chronic illnesses (such as chronic hypertension, pregestational diabetes, renal, pulmonary, and thyroid disease), those who were HIV positive, funisitis and fetuses with malformations incompatible with life. Women with incomplete medical records were excluded.

### 2.2 Procedure

Consecutive cases of patients with HCA were obtained from the Pathology Department records; after that, the clinical characteristics and perinatal outcomes until seven days after birth were obtained from the clinical records. As part of prenatal care, all women received monthly prenatal visits with an obstetrician until week 32, every 2 weeks from 32 to 37 weeks of gestation, and weekly thereafter. Patients with a history of cervical cerclage had cervical-vaginal cultures every month. In case of clinical suspicion of chorioamnionitis, the pregnancy was immediately interrupted, and double antibiotic regimen with ampicillin and gentamicin was initiated. After cesarean delivery, clindamycin was added. All placentas (term and preterm deliveries) were sent for histopathological analysis during the study period.

Women with primary HCA were divided into two groups: group 1 women with CCA and group 2 women with SCCA as reported in clinical records.

CCA was defined as the diagnosis of suspected intraamniotic infection, and maternal temperature is greater than or equal to 39.0°C, or when the maternal temperature is 38.0–38.9°C, and one additional clinical risk factor is present (21).

SCCA was defined as inflammation of the placenta without any clinical signs of chorioamnionitis as previously defined (11).

Maternal leukocytosis was defined as white blood cells greater than 15,000/mm3 (25).

Fetal tachycardia is an abnormal baseline heart rate during 10 min or more above 160 bpm (26).

Purulent cervical discharge is purulent or foul smelling amniotic fluid or cervical discharge (27).

Secondary; placentas were reclassified by a Perinatal Pathologist according with the proposed classification of the Amsterdam Placental Workshop Group Consensus in stage 1, 2 and 3 and grade 1 and 2 (13).

### 2.3 Primary outcomes

The primary outcome was to compare the risk of perinatal outcomes in women with HCA with CCA and SCCA as reported in clinical records, and the additional outcome was to compare the prevalence and risk of adverse perinatal outcomes according with the Classification by Amsterdam Placental Workshop Group Consensus. The adverse perinatal outcomes included:

PTB: any birth before 37 completed weeks of gestation (28).

Neonatal sepsis was defined as the presence of both infection (positive cultures) and systemic inflammatory response (29, 30). The study included only early neonatal sepsis, defined as an infection that occurs within the first 72 h of life and usually reflects vertical transmission.

Stillbirth was defined as within WHO criteria as a dead fetus of 1,000 g or more at birth, or after 28 completed weeks of gestation, or attainment of at least 35 cm crown-heel length (31, 32).

Early neonatal death is defined as deaths between 0 and 7 completed days of birth (33).

Premature rupture of membranes (PROM) was defined as membrane rupture before labor, membranes rupture that occurs before 37 weeks of gestation is referred to as preterm PROM (34).

Prolonged rupture of membranes (Prolonged PROM) is considered when the duration exceeds 18 h prior to delivery (35).

### 2.4 Data analysis

A software SPSS database was used to capture data [SPSS version 22.0 for Windows (SPSS Inc. Chicago, IL)]. Demographic and clinical variables were analyzed with descriptive statistics. Student’s t-test was used for the analysis of continuous variables. A *p* value of less than 0.05 was considered statistically significant. To test the association between placental pathology and perinatal evolution, the Pearson’s chi-square test for dichotomous variables and/or Fisher’s exact test was performed. The relative risk was calculated with a 95% confidence interval.

### 2.5 Sample size

The sample size was calculated using recommendations for sample size estimation for difference of proportions in neonatal sepsis with an estimation of 20% in SCCA and 50% in CCA with a 95% confidence level, and statistical power of 80%, a sample size of 39 participants was required (16, 36). We decided to include all patients with HCA who fulfill the inclusion criteria during the period of study.

## 3 Results

### 3.1 Patients characteristics

There were 404 cases with the diagnosis of HCA during the study period. Ninety-three patients were excluded due to incomplete medical record with a total of 311 cases with HCA. There were 41 cases of CCA and 270 cases of SCCA. The mean age of the women was 28.6 ± 7.2 years, ranging from 14 to 47 years, with 113 (36.3%) primiparous. Vaginal delivery was performed in 84 cases (27%), while cesarean delivery was performed in 227 cases (73%). The prevalence of cesarean sections at this institution is notably elevated, primarily attributed to the specific patient demographic that seeks prenatal care at this facility.

Of the 311 patients, 175 (56%) had stage 1 HCA, 79 (25%) had stage 2 chorioamnionitis, and 57 (19%) had stage 3 chorioamnionitis. Two hundred thirteen infants (67.8%) were preterm. The results showed no significant difference in maternal age, placental weight, and gravidity between the CCA and SCCA groups (*p* > 0.05). However, a statistically significant difference was found in fetal weight and gestational age at birth between the two groups (*p* < 0.05). The mean fetal weight was significantly lower in the CCA group (1,492 ± 854 grams) than in the SCCA group (1864 ± 876 grams). The mean gestational age at birth was also significantly lower in the CCA group (30.2 ± 5.4 weeks) than in the SCCA group (32.5 ± 5.1 weeks) (see Table 1).

### 3.2 Risk factors

One hundred and eighty-seven patients (60.1%) had no relevant past medical history, 46 (14.7%) had diabetes mellitus or carbohydrate intolerance, 42 (13.5%) had hypertensive disease of pregnancy, 33 (10.6%) had cervical insufficiency, 17 (5.4%) had morbid obesity, 16 (5.1%) had heart disease, 10 (3.2%) had autoimmune disease, 5 (1.6%) had mild renal disease and 43 (13.8%) patients had more than one concomitant disease. One hundred fifty-five patients (50%) had premature rupture of membranes (PROM); seventy-nine (25.4%) patients had prolonged PROM. Only 33 (10.6%) of women with PROM received a conservative treatment.

**Table 1:** Clinical characteristics of women with clinical chorioamnionitis and sub-clinical chorioamnionitis.

	CCA Mean ± SD (*n* = 41)	SCCA Mean ± SD (*n* = 270)	Total	p*
Maternal age (years)	28.9 ± 7.8	28.5 ± 7.1	28.6 ± 7.2	0.781
Placental weight (g)	314 ± 138	365 ± 149	358 ± 148	0.042
Gravidity	2.76 ± 2.0	2.42 ± 1.4	2.47 ± 1.5	0.204
Fetal weight (g)	1,492 ± 854	1864 ± 876	1815 ± 881	0.012
Gestational age at birth (weeks)	30.2 ± 5.4	32.5 ± 5.1	32.2 ± 5.2	0.007

*T student’s test. CCA = Clinical Chorioamnionitis, SCCA = Sub-clinical Chorioamnionitis, SD = standard deviation.

Table 2 shows that cervical insufficiency, PROM, Prolonged PROM, maternal leukocytosis, fetal tachycardia, and purulent cervical discharge are all significantly increased in women with CCA.

**Table 2:** Risk factors of women with clinical chorioamnionitis and sub-clinical chorioamnionitis.

Risk factor	CCA (*n* = 41)	SCCA (*n* = 270)	Total (*n* = 311)	Relative risk 95% CI	p*
PROM	30 (73.2%)	125 (46.3%)	155(49.8%)	3.16 (1.52–6.57)	0.001
Cervical insufficiency	8 (19.5%)	25 (9.3%)	33 (10.6%)	2.37 (1.0–5.70)	0.04
Prolonged PROM	16 (39%)	63 (23.3%)	79 (25.4%)	2.1 (1.05–4.18)	0.03
Fever >39°C	28 (68.2%)	0 (0%)	28 (9%)	183 (25–1,311)	0.0001
Fever >38°C	41 (100%)	0 (0%)	41 (13.1%)	265 (37–1880)	0.0001
Maternal leukocytosis	24 (58.5%)	73 (27%)	97(31.2%)	3.8 (1.93–7.49)	0.001
Fetal tachycardia	6 (14.6%)	9 (2.9%)	15 (4.8%)	4.97 (1.66–14.81)	0.002
Purulent cervical discharge	20 (48.8%)	17 (6.3%)	37 (11.9%)	14.1 (6.46–31.07)	0.0001

CCA = Clinical Chorioamnionitis, SCCA = Sub-clinical Chorioamnionitis, PROM = Preterm Premature Rupture of Membranes *Chi square test.

### 3.3 Adverse perinatal outcomes

Table 3 shown the results on adverse perinatal outcomes between women with CCA and SCCA. The CCA group had a significantly higher risk of PTB, fetal weight less than 1,500 g, and neonatal sepsis compared to the SCCA group. However, there was no significant difference in the risk of stillbirth or early neonatal death between the two groups.

**Table 3:** Adverse perinatal outcomes of women with clinical chorioamnionitis and sub-clinical chorioamnionitis.

Perinatal outcome	CCA (*n* = 41)	SCCA (*n* = 270)	Total (*n* = 311)	Relative risk 95%CI	p*
Preterm birth	34 (82.9%)	177 (65.6%)	211(67.8%)	2.55 (1.08–5.97)	0.026
Fetal weight < 1,500 g	26 (63.4%)	108 (40%)	134 (43.1%)	2.6 (1.1–5.13)	0.005
Neonatal sepsis	20 (48.8%)	51 (18.8%)	71 (22.9%)	4.07 (2.05–8.06)	0.0001
Stillbirth	9 (22%)	35 (13%)	44(14.1%)	1.88 (0.83–4.28)	0.124
Early neonatal death	0 (0%)	11(4.1%)	11 (3.5%)	0.93 (0.86–1.03)	0.188

CCA = Clinical Chorioamnionitis, SCCA = Sub-clinical Chorioamnionitis, PROM = Preterm Premature Rupture of Membranes *Chi square test.

Table 4 shown a comprehensive comparison of adverse perinatal outcomes based on the staging HCA. The prevalence of CCA significantly differs among the groups, with a higher prevalence observed in cases of stage 2 and 3. Additionally, an increased prevalence of CCA, preterm births, PROM, prolonged PROM, fetal weight below 1,500 g, neonatal sepsis and stillbirth as the staging of HCA increases.

**Table 4:** Comparison of perinatal outcomes based on the staging of chorioamnionitis histopathology according to Amsterdam placental workshop group consensus classification.

Outcome	Stage 1 (*n* = 175)	Stage 2 (*n* = 79)	Stage 3 (*n* = 57)
CCA	10 (5.7%)	10 (12.7%)	21 (36.8%)^b***, c**^
Preterm birth	109 (62.2%)	57 (72.1%)	45 (78.9%)^b*^
PROM	71 (40.5%)	41(51.8%)	43 (75.4%)^b***, c**^
Prolonged PROM	30 (17.1%)	27 (34.1%)^a**^	22 (38.5%)^b***^
Fetal weight < 1,500 g	58 (33.1%)	38 (48.1%)^a*^	38 (66.6%)^b***, c*^
Neonatal sepsis	23 (13.1%)	23 (29.1%)^a**^	25 (43.8%)^b***^
Stillbirth	20 (11.4%)	10 (12.6%)	14 (24.5%)^b*^
Early neonatal death	6 (3.4%)	4 (5%)	1 (1.7%)

CCA = Clinical Chorioamnionitis, PROM = Preterm Premature Rupture of Membranes. ^a^Stage 1 Vs. Stage 2; ^b^Stage 1 Vs. Stage 3; ^c^Stage 2 Vs. Stage 3.*p < 0.05, **p < 0.001 Pearson’s Chi-squared test or Fisher’s exact test.

Table 5 provides a detailed analysis of the relative risk associated with grade 2 of HCA in relation to the development of adverse perinatal complications, comparing it with grade 1 of HCA. Grade 2 HCA was significantly associated with clinical chorioamnionitis, preterm birth, immature birth, PROM, fetal weight below 1,500 g, neonatal sepsis, stillbirth, and early neonatal death.

**Table 5:** Relative risk of severe histological chorioamnionitis for the development of perinatal complications according to Amsterdam placental workshop group consensus classification.

Outcome	Grade 1 (*n* = 254)	Grade 2 (*n* = 57)	p*	RR (IC95%)
CCA	20 (7.8%)	21 (36.8%)	0.001	4.6 (2.7–8.03)
Preterm birth	166 (65.3%)	45 (78.9%)	0.047	1.98 (1–3.9)
PROM	112 (44%)	43 (75.4%)	0.001	3.8 (2.02–7.4)
Prolonged PROM	58 (22.8%)	21 (36.8%)	0.04	1.61 (1.07–2.4)
Fetal weight < 1,500 g	96 (37.7%)	38 (66.6%)	0.001	3.2 (1.7–6.03)
Neonatal sepsis	46 (18.1%)	25 (43.8%)	0.001	3.5 (1.9–6.4)
Stillbirth	30 (11.8%)	14 (24.5%)	0.01	2.4 (1.1–4.9)
Early neonatal death	10 (3.9%)	1 (1.7%)	0.4**	0.5 (0.007–3.2)

CCA = Clinical Chorioamnionitis, PROM = Preterm Premature Rupture of Membranes.*Pearson’s Chi-squared test, **Fisher’s exact test.

## 4 Discussion

In this study, it was demonstrated that the risk of perinatal complications is higher in women with HCA, as expected, however a substantial number of women with SCCA also experienced perinatal complications. Therefore, the histopathological examination of the placenta is of great importance among women who exhibit one or more of the following characteristics towards the end of pregnancy: PROM, Prolonged PROM, preterm birth, maternal fever, maternal leukocytosis, fetal tachycardia, and purulent cervical discharge.

Chorioamnionitis can be defined clinically, histologically, or microbiologically. However, it is important for clinicians to be aware that even in the absence of microbiological evidence, acute histologic chorioamnionitis can also occur as a result of sterile intra-amniotic inflammation, which is induced by danger signals released under cellular stress, injury, or death (37, 38); perhaps the most important of them is the high intra-uterine volume, inevitable in term pregnancies. There is a significant number of women who show histological evidence of chorioamnionitis but exhibit subclinical progression, likely because they are in the early stages of the infectious process. The classic clinical presentation of chorioamnionitis is still commonly observed. However, there are preceding phenomena, such as intraamniotic infection, that can lead to the termination of pregnancy without the development of overt clinical symptoms of chorioamnionitis (39).

In the present study, there were 11 early neonatal deaths (3.5%) attributed to prematurity and/or neonatal sepsis. Considering the high rate of neonatal sepsis (22%) and preterm birth (67.8%), there was a trend of low early neonatal death rate, which can be attributed to the fact that only 43% of neonates had a weight < 1,500 g, the availability of infrastructure, economic and human resources in neonatal intensive care unit and were similar to those reported in our institution by Rivera-Rueda et al. (40). In comparison, a cohort study conducted in a tertiary center in China, focusing on pregnancies with preterm PROM before 34 weeks of gestation managed conservatively, reported a 17.8% incidence of clinically diagnosed chorioamnionitis (CCA). The neonatal mortality rate was 7.4%, and major neonatal complications occurred in 40% of cases (41). Furthermore, another study examining premature rupture of membranes before 35 weeks of gestation found that chorioamnionitis was the most common maternal complication, occurring in 34.7% of cases, neonatal sepsis was observed in 12% of patients, and the perinatal mortality rate was 21.5% for the group over 24 weeks of gestation and 76.5% for the group under 24 weeks of gestational age (42). The clinical manifestations observed in these studies highlight the significant impact of chorioamnionitis on both maternal and neonatal outcomes in cases of preterm rupture of membranes.

If, well, in our study, the incidence of neonatal deaths was relatively low (3.5%), the incidence of stillbirth was four times higher (14.1%) and more frequent in the CCA (22%) than in the SCCA (13%) group. Notably, within the stillbirth category, the incidence was more pronounced in the CCA group at 22%, compared to the SCCA group at 13%. This disparity may be elucidated by the documented association between chorioamnionitis and stillbirths, as highlighted in previous studies; for instance, Pinar et al. conducted a study involving 518 singleton stillbirths, revealing that the most frequent placental abnormalities associated with stillbirth were acute chorioamnionitis of the free membranes (30%) or chorionic plate (23%). Moreover, they reported inflammatory lesions, such as massive perivillous fibrin deposition, in 9.2% of stillbirth cases (43). Likewise, Man et al. examined 946 stillbirths with placental histological evaluation as part of the autopsy process. Their findings indicated ascending infection as the cause of death in 19% of cases, while 2% exhibited inflammatory placental abnormalities (44). These findings collectively suggest a compelling correlation between chorioamnionitis and stillbirths, underscoring the importance of understanding and addressing future research on these factors in maternal and neonatal care. The clinical manifestations had a notable impact on the clinical course, leading to an increased risk of preterm delivery, PROM, and consequently a higher rate of preterm births with neonates weighing less than 1,500 g, as was observed in the group of women with CCA. Interestingly, a high frequency of these clinical manifestations was also found among women with SCCA, which could be contradictory but could be explained by the definition of CCA and SCCA. Therefore, future research could be directed toward identifying an accessible and efficient method to diagnose this group of patients early, allowing for timely interventions and improved outcomes.

Having a diagnostic protocol that incorporates amniotic fluid cultures and systemic inflammation markers could enhance the detection rate of SCCA. According to Galaz, amniotic fluid analysis of women with preterm CCA and positive cultures revealed several characteristics: (1) presence of abundant neutrophils along with viable and non-viable bacteria, (2) neutrophils engaged in phagocytosis, (3) formation of neutrophil extracellular traps by neutrophils, (4) increased numbers of neutrophils, monocytes/macrophages, and CD4+ T cells, and (5) high expression of IL-1β by neutrophils and monocytes/macrophage (45). Since histopathological examination is time-consuming and impractical for acute cases seeking care at clinics and hospitals, alternative diagnostic tools are needed. Both procalcitonin (PCT) and C-reactive protein (CRP) show promise in predicting subclinical intrauterine infection in pregnant women with PROM before 34 weeks of gestation. PCT and CRP have good potential for diagnostic prediction, with PCT being particularly applicable to pregnant women between 28 and 33 + 6 weeks of gestation with PROM (46).

Uterine activity and premature PROM are important risk factors for chorioamnionitis. However, physiological changes that occur during pregnancy can affect the interpretation of inflammatory biomarkers and white blood cell counts, making it challenging to rely solely on clinical suspicion (47). The neutrophil-to-lymphocyte ratio (NLR) has been investigated as a potential predictor of histological chorioamnionitis, an elevated NLR was associated with a five-fold increased risk of HCA in women who delivered prematurely without signs or symptoms of infection. Additionally, NLR levels were higher at the time of preterm labor compared to the first trimester. These findings suggest that NLR could serve as a useful biomarker for identifying women at risk of chorioamnionitis and potentially aid in clinical decision-making (48). Imaging of the fetal immune system, in particular the thymus and the spleen, and the placenta may give valuable information antenatally regarding the diagnosis of fetal inflammatory response (49).

A significant strength of the study was the confirmation of chorioamnionitis through histopathological examination, as it provided robust support for the study variable. Like Fahmi’s study, which demonstrated an association between HCA grade 2 and early preterm birth, while HCA grade 1 appeared to correlate with full-term deliveries (50), our investigation revealed a statistically significant connection between Grade 2 HCA and various adverse outcomes compared to HCA grade 1, including clinical chorioamnionitis, preterm birth, premature rupture of membranes (PROM), fetal weight below 1,500 g, and neonatal sepsis, but, was not significative differences for early neonatal death, that could be explained by the low frequency of this adverse outcome and in consequence the lack of statistical power.

An important limitation of the study was its retrospective design, which resulted in a lack of uniformity in data collection from the included women. Some patients had inflammation and infection markers such as erythrocyte sedimentation rate, CRP, and PCT, but these could not be analyzed consistently due to the lack of uniformity in data availability. Furthermore, relevant clinical data such as the number of vaginal examinations performed were not reliably recorded, despite this being a well-described risk factor for chorioamnionitis in the literature (51). This limitation may have impacted the ability to fully assess and analyze the association between certain clinical factors and the occurrence of chorioamnionitis.

It is important to acknowledge these limitations, as they may have influenced the overall findings and interpretation of the study results. Furthermore, future research could concentrate on identifying potential risk factors associated with SCCA in large cohort studies. Additionally, it could assess the potential role of HCA as a biomarker, illustrating fetal-maternal defense functions following an injury with both short and long-term implications for the mother and the fetus”.

## 5 Conclusion

Two-thirds of patients with HCA had a preterm newborn, while almost half experienced PROM. Interestingly, clinical manifestations were observed in only 13 % of patients with HCA. However, it is noteworthy that patients with SCCA also faced a high risk of perinatal complications. Frequency of stillbirth and early neonatal death was increased in women with HCA.

## Data availability statement

The raw data supporting the conclusions of this article will be made available by the authors, without undue reservation.

## Ethics statement

This study was approved by Institutional Review Board of Instituto Nacional de Perinatología Isidro Espinosa de los Reyes; ID number: CEI-RETRO-01-2023, approved May 2023. This study was conducted in accordance with the local legislation and institutional requirements. Written informed consent for participation was not required from the participants or the participants’ legal guardians/next of kin because in retrospective studies, the data obtained from clinical records is not possible to get written informed consent, only the IRB reviewed and approved the research.

## Author contributions

AO-O, ER-M, and RF-D: conceptualization. AO-O, ER-M, MP-V, and RF-D: methodology and validation. AO-O and ER-M: software, data curation, writing—original draft preparation, and writing—review and editing. AO-O, MP-V, and ER-M: formal analysis. AO-O: investigation, resources, and visualization. ER-M and RF-D: supervision. All authors contributed to the article and approved the submitted version.
